# 35. Health-related quality of life in COVID-19 survivors after 12 months, a prospective cohort study

**DOI:** 10.1093/ofid/ofab466.035

**Published:** 2021-12-04

**Authors:** Sebastiaan Siegerink, Marië Nijpels, Sander Albers, Frédérique Jurgens, Felix K Pettai, Laura Samwel, Joost Vanhommerig, Paul Bresser, Marieke de Regt, Birit Broekman, Kees Brinkman

**Affiliations:** OLVG Amsterdam, Amsterdam, Noord-Holland, Netherlands

## Abstract

**Background:**

The long-term effects of COVID-19 are still unknown. This study aims to assess the impact of COVID-19 among survivors after one year.

**Methods:**

All confirmed COVID-19 cases who presented at OLVG hospital in Amsterdam during the first wave of the COVID-19 pandemic were invited to participate in our prospective observational cohort study. The participants were divided into three subgroups: patients not admitted, admitted to the general ward and admitted to the ICU. Questionnaires were sent at 3, 6 and 12 months after presentation. We used the Research and Development – 36-item health survey, the Hospital Anxiety and Depression Scale and the PTSS Checklist for DSM-5. We compared the RAND-36 scores at the timepoints with a Dutch healthy control population in 2020 and between the three subgroups using the Kruskal-Wallis test and the Mann-Whitney U test.

**Results:**

Of the 466 confirmed cases, 75 patients died of COVID-19, 64 patients were lost to follow up and 12 patients were excluded because they were unable to complete the questionnaires due to mental illness or cognitive impairment, they moved back to their home country or refused to participate. Of the remaining 315 patients, 182 (57.8%) completed the questionnaires at 3 months. Subsequently, 163 patients provided informed consent for follow up. At 6 and 12 months, 98 (60.1%) and 131 (80.4%) completed the survey. The average score of all domains at 3 months was 58, compared to 79 at twelve months and 81 in the control group. There was a statistically significant increase from 3 and 12 and 6 and 12 months (figure 1). At twelve months participants recovered to levels of the healthy control group (N=459), except for the ICU group, who still experienced bodily pain and decreased physical function. The improvement was most noticeable in the domains of social functioning, role limitations – physical and role limitations – emotional. The percentage of patients with abnormal total HADS scores (cutoff at 16) and PCL5- scores (cutoff at 33) at 3 months decreased from 27.8 to 22.1% and 18.9 to 7.6% at 12 months, respectively (figure 2 and 3).

Figure 1. RAND-36: Health-related quality of life after COVID-19 of all patients.

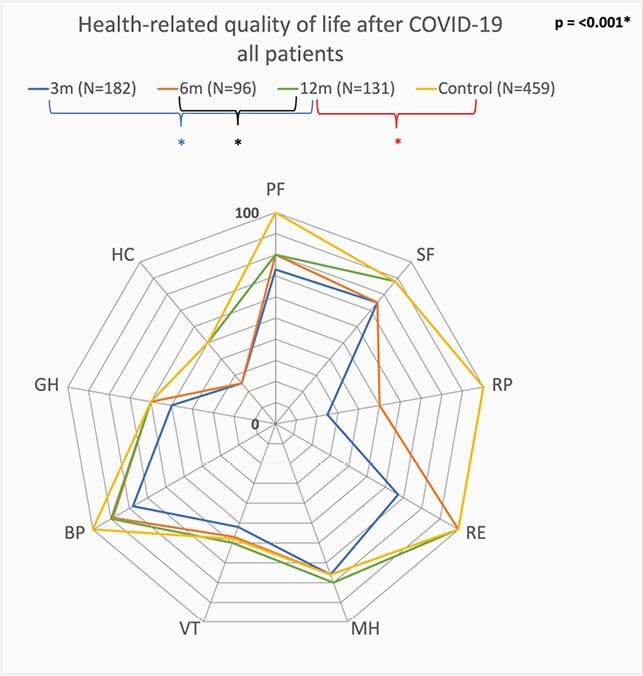

Blue line is after 3 months, orange line is after 6 months, green line is after 12 months, yellow line is healthy control. The p-value in the right-upper corner shows statistical significant difference between all total scores, the asterisks indicate significance between groups. PF = physical functioning; SF = social functioning; RP = role limitations–physical; RE = role limitations–emotional; MH = mental health; VT = vitality; BP = pain; GH = general health; HC = health change.

Figure 2

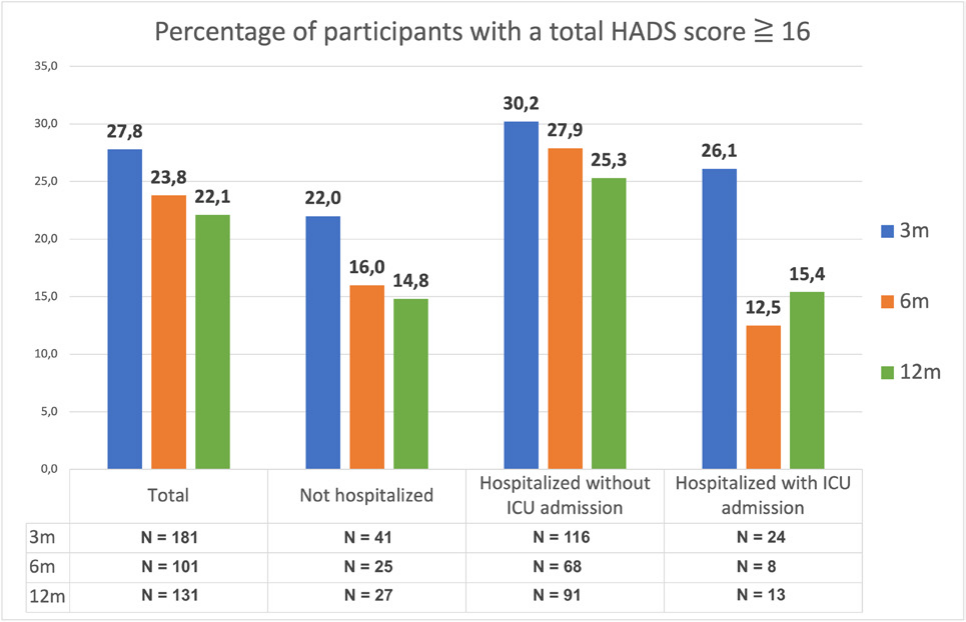

The blue column is after 3 months, the orange after 6 months and the green after 12 months. The numbers above the columns are percentages per group.

Figure 3

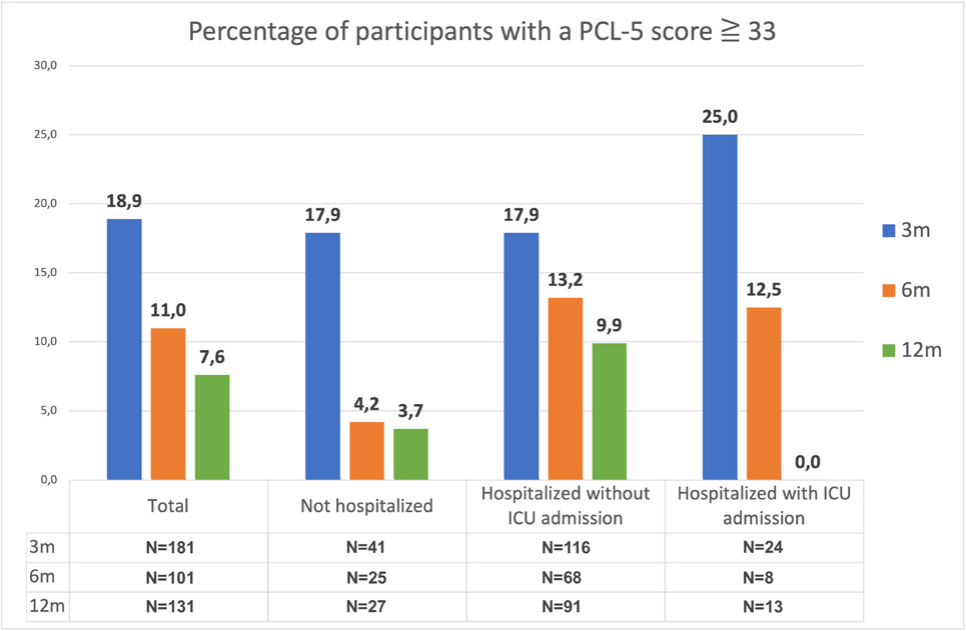

The blue column is after 3 months, the orange after 6 months and the green after 12 months. The numbers above the columns are percentages per group.

**Conclusion:**

Although, COVID-19 may cause a decreased health-related quality of life and impaired mental health, this study shows important recovery up to normal levels after one year.

**Disclosures:**

**All Authors**: No reported disclosures

